# Colonic Lymphangioma as a Lead Point for Adult Ileocecal Intussusception: A Case Report

**DOI:** 10.7759/cureus.106461

**Published:** 2026-04-05

**Authors:** Hind Y Naseem, Bayan I Bokhari

**Affiliations:** 1 Colorectal Surgery, King Abdulaziz Medical City, Jeddah, SAU

**Keywords:** adult intussusception etiology, bowel obstruction, colonic lymphangioma, ileocecal intussusception, laparoscopic colorectal surgery, rare colonic tumor

## Abstract

Colonic lymphangiomas are rare benign malformations of the lymphatic system and are particularly uncommon within the gastrointestinal tract. In adults, intussusception is an uncommon condition and is often associated with an identifiable structural lead point.

We report a rare case of an adult patient presenting with symptoms of bowel obstruction who was found to have an ileocecal intussusception caused by a colonic lymphangioma. Imaging suggested the presence of an underlying lesion, and a definitive diagnosis was established following surgical resection and histopathological examination.

This case highlights the importance of considering rare benign lesions, such as lymphangioma, in the differential diagnosis of adult intussusception. Early recognition and surgical management are essential for both diagnosis and treatment.

## Introduction

Colonic lymphangioma is an exceedingly rare benign tumor arising from lymphatic vessels. Although lymphangiomas are more commonly encountered in the head, neck, and mesentery, the involvement of the gastrointestinal tract, particularly the colon, is uncommon [1,2]. These lesions are typically diagnosed in middle-aged adults and show a slight male predominance.

The exact pathogenesis of lymphangiomas remains uncertain. In adults, they are believed to result from acquired disturbances in lymphatic drainage, which may occur secondary to chronic inflammation, previous surgical interventions, trauma, or radiation exposure [2]. Among abdominal lymphangiomas, colonic involvement represents a very small proportion of reported cases and is therefore considered a rare clinical entity [3,4].

Most colonic lymphangiomas remain asymptomatic and are often detected incidentally during endoscopic or radiologic evaluation. However, symptomatic cases may present with nonspecific gastrointestinal manifestations.

Here, we report a rare case of adult colonic lymphangioma presenting with intussusception.

## Case presentation

A 32-year-old female patient with no significant past medical or surgical history initially presented to an outside hospital in January 2025 with acute abdominal pain and clinical features suggestive of bowel obstruction. Cross-sectional imaging, including contrast-enhanced computed tomography (CECT) and magnetic resonance imaging (MRI), demonstrated ileocecal intussusception associated with cecal and appendiceal wall thickening. She was admitted for conservative management, during which her symptoms improved and bowel function resumed. No surgical intervention was required at that time, and she was subsequently discharged.

In July 2025, she presented to our institution with a one-month history of intermittent right lower quadrant abdominal pain. The pain initially began in the periumbilical region before localizing to the right lower quadrant and was associated with nausea, vomiting, and constipation. She denied fever, weight loss, gastrointestinal bleeding, or changes in appetite. There was no significant personal or family history of malignancy. On physical examination, the patient was hemodynamically stable, and abdominal examination was unremarkable.

**Figure 1:**
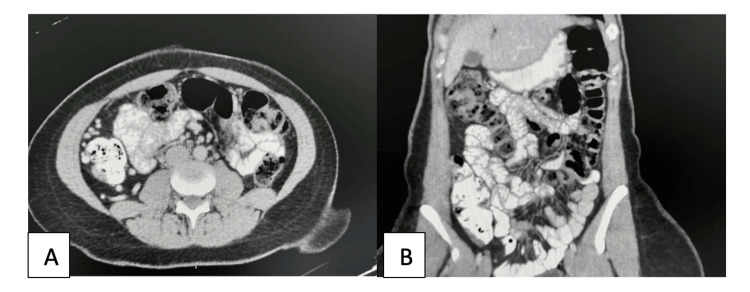
Contrast-enhanced CT scan of the abdomen (A) Axial contrast-enhanced CT image demonstrating eccentric thickening of the inferior cecal wall (B) Coronal view showing focal mural thickening at the ileocecal region without evidence of bowel obstruction or active intussusception

A contrast-enhanced CT scan of the abdomen and pelvis revealed eccentric thickening of the inferior wall of the cecum, measuring approximately 6 mm in maximal thickness and extending over a length of approximately 2 cm (Figures 1A, 1B). There was no radiologic evidence of recurrent intussusception, bowel obstruction, ascites, or significant lymphadenopathy. The appendix appeared normal. An incidental right adnexal hypodense lesion consistent with a functional ovarian cyst was also noted.

**Figure 2:**
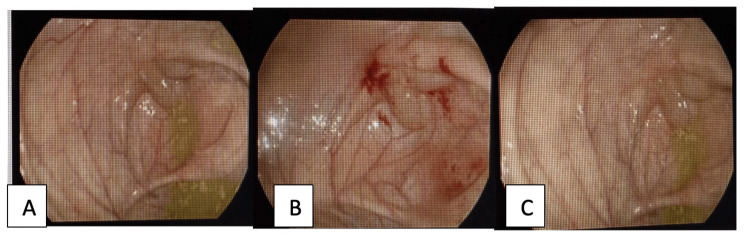
Colonoscopy images (A) Subepithelial polypoid lesion at the appendiceal orifice (B) Close-up view showing intact but mildly erythematous mucosa following biopsy (C) Additional view demonstrating multilobulated configuration

Colonoscopy was performed for further evaluation and revealed a multilobulated polypoid lesion measuring approximately 10 mm at the appendiceal orifice with an overall benign-appearing mucosal surface (Figures 2A-2C). Given the mucinous appearance and suspicion of submucosal pathology, biopsies were obtained from the surrounding mucosa. Histopathological analysis demonstrated normal colonic mucosa without evidence of dysplasia, granuloma, infection, or malignancy.

Given the patient’s prior presentation with ileocecal intussusception, which in adults is strongly associated with a pathological lead point and raises concern for underlying malignancy, along with persistent symptoms and indeterminate preoperative findings, elective laparoscopic cecectomy was performed without intraoperative complications.

**Figure 3:**
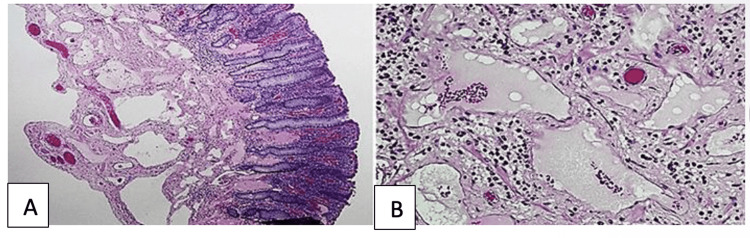
Histopathology examination of the resected specimen (A) Low-powered view showing multiple dilated lymphatic channels within the submucosa (H&E stain) (B) High-powered view demonstrating cystically dilated spaces lined by a single layer of flattened endothelial cells, consistent with a cystic lymphangioma

Gross examination of the resected specimen revealed a submucosal multiloculated cystic lesion measuring 1.5 × 0.5 cm within the cecal wall containing mucinous material. Histopathological evaluation confirmed the diagnosis of cecal lymphangioma (Figures 3A, 3B). The appendix was unremarkable, and surgical margins were clear. Five reactive lymph nodes were identified with no evidence of malignancy. The mesoappendix demonstrated benign fibro-fatty tissue.

The postoperative course was uneventful. The patient recovered well and was discharged on postoperative day two. Follow-up evaluation revealed no postoperative complications.

## Discussion

Colorectal lymphangioma is an exceedingly rare benign lesion, most commonly diagnosed between the fourth and sixth decades of life, with a slight male predominance, reported at approximately 1.4-1.6:1 [1,2]. In adults, lymphangiomas are thought to arise secondary to acquired disturbances in lymphatic drainage, potentially related to chronic inflammation, previous surgical procedures, or radiation exposure [2]. Among abdominal lymphangiomas, the majority are located in the mesentery and retroperitoneum, while involvement of the colonic wall is exceptionally uncommon, accounting for approximately 0.7% of cases [3,4].

The most frequently reported sites of colorectal lymphangiomas include the transverse colon, ascending colon, and cecum, with most presenting as solitary lesions [1,2]. Lesion size is variable, with reported diameters reaching up to 23 cm [1,2]. Histologically, lymphangiomas are classified into simple (capillary), cavernous, and cystic types based on the morphology of the lymphatic spaces [5]. Cystic lymphangiomas represent the most common subtype, accounting for approximately 70% of cases, with nearly 80% demonstrating a multilocular configuration [1].

Clinically, small colonic cystic lymphangiomas are often asymptomatic. When symptoms occur, they are typically nonspecific and depend on lesion size and location, most commonly presenting with abdominal pain, with or without hematochezia, diarrhea, or constipation [2]. However, several complications have been reported, including secondary infection [6], intussusception [7,8], anemia [9,10], and protein-losing enteropathy [11,12]. To date, malignant transformation of colonic lymphangiomas has not been reported.

Ultrasonography is frequently the initial imaging modality in symptomatic abdominal presentations. On ultrasound, cystic lymphangiomas characteristically appear as multiloculated cystic masses containing anechoic fluid [8]. CT imaging is particularly useful for assessing disease extent and excluding complications such as obstruction or intussusception [9,13,14]. MRI offers superior soft tissue contrast and improved delineation of the cystic nature of these lesions [15].

Endoscopically, cystic lymphangiomas typically present as smooth, subepithelial lesions with intact mucosa. Endoscopic ultrasonography (EUS) has been widely reported as valuable in both definitive and differential diagnosis [2,10,14,16]. On EUS, these lesions are usually confined to the submucosal layer, demonstrating well-defined margins with anechoic cystic spaces and preservation of the muscularis propria. These findings aid in differentiating colonic lymphangiomas from other submucosal tumors, such as gastrointestinal stromal tumors, leiomyomas, schwannomas, lymphoma, and neuroendocrine tumors, which are typically hypoechoic [17,18].

Based on these imaging characteristics, some authors suggest that endoscopic biopsies may be unnecessary and may increase the risk of infection in multicystic lesions [19]. Management remains individualized and depends on lesion size, symptoms, and diagnostic certainty. Asymptomatic lesions smaller than 2 cm may be managed conservatively [19]. Endoscopic polypectomy has been successfully performed for lesions measuring 2-3.5 cm [6,8], with some reports describing resection of lesions up to 5 cm in diameter [20].

However, when endoscopic management is technically challenging, when malignancy cannot be excluded, or when complications such as intussusception occur, surgical resection is recommended [14]. Adult colonic lymphangioma-related intussusception is rare and most commonly involves the cecum as the lead point. Even small lesions may precipitate intussusception or progressive local complications, leading several authors to advocate surgical resection as definitive management.

Our case contributes to the limited body of literature describing adult colonic lymphangioma presenting with intussusception and highlights several important clinical considerations, including the potential for recurrence after conservative management, the limitations of imaging and endoscopic biopsy in establishing a definitive diagnosis, and the role of surgical resection as both a diagnostic and therapeutic modality.

## Conclusions

Colonic lymphangioma is a rare benign lesion that may present with nonspecific clinical and radiologic findings, often leading to diagnostic uncertainty. When located in the cecum or at the appendiceal orifice, it may act as a lead point for intussusception even in young adults. This case highlights the limitations of preoperative imaging and endoscopic biopsy in establishing a definitive diagnosis. Surgical resection, therefore, remains both a diagnostic and therapeutic approach, particularly in symptomatic patients or when malignancy cannot be confidently ruled out.
